# Trends in biodiversity and habitat quantification tools used for market‐based conservation in the United States

**DOI:** 10.1111/cobi.13349

**Published:** 2019-07-10

**Authors:** Scott J. Chiavacci, Emily J. Pindilli

**Affiliations:** ^1^ Science and Decisions Center U.S. Geological Survey 12201 Sunrise Valley Drive Reston VA 20192 U.S.A.

**Keywords:** biodiversity offset, compensatory mitigation, conservation bank, ecolabel, habitat exchange, payments for ecosystem services, banco de conservación, compensación de biodiversidad, eco‐etiqueta, intercambio de hábitat, mitigación compensatoria, pago por servicios ambientales

## Abstract

Market‐based conservation mechanisms are designed to facilitate the mitigation of harm to and conservation of habitats and biodiversity. Their potential is partly hindered, however, by the quantification tools used to assess habitat quality and functionality. Of specific concern are the lack of transparency and standardization in tool development and gaps in tool availability. To address these issues, we collected information via internet and literature searchers and through conversations with tool developers and users on tools used in U.S. conservation mechanisms, such as payments for ecosystem services (PES) and ecolabel programs, conservation banking, and habitat exchanges. We summarized information about tools and explored trends among and within mechanisms based on criteria detailing geographic, ecological, and technical features of tools. We identified 69 tools that assessed at least 34 species and 39 habitat types. Where tools reported pricing, 98% were freely available. More tools were applied to states along the U.S. West Coast than elsewhere, and the level of tool transferability varied markedly among mechanisms. Tools most often incorporated conditions at numerous spatial scales, frequently addressed multiple risks to site viability, and required 1–83 data inputs. Most tools required a moderate or greater level of user skill. Average tool‐complexity estimates were similar among all mechanisms except PES programs. Our results illustrate the diversity among tools in their ecological features, data needs, and geographic application, supporting concerns about a lack of standardization. However, consistency among tools in user skill requirements, incorporation of multiple spatial scales, and complexity highlight important commonalities that could serve as a starting point for establishing more standardized tool development and feature‐incorporation processes. Greater standardization in tool design may expand market participation and facilitate a needed assessment of the effectiveness of market‐based conservation.

## Abstract 2

*Article impact statement*: Improving biodiversity and habitat quantification tool availability, standardization, and transparency benefits market‐based conservation.

## Abstract (es)

Tendencias en la Biodiversidad y en las Herramientas de Cuantificación de Hábitat Usadas para la Conservación Basada en el Mercado en los Estados Unidos

**Resumen**

Los mecanismos de conservación basada en el mercado están diseñados para facilitar la mitigación del daño y la conservación de los hábitats y la biodiversidad. Sin embargo, el potencial de estos mecanismos está parcialmente reducido por las herramientas de cuantificación usadas para evaluar la calidad y funcionalidad del hábitat. Son de preocupación específica la falta de transparencia y la estandarización del desarrollo de herramientas y los vacíos en la disponibilidad de las herramientas. Para tratar estos temas recolectamos información por medio del internet y los buscadores de literatura y a través de conversaciones con los desarrolladores y usuarios de las herramientas utilizadas en los mecanismos de conservación en los Estados Unidos, como la eco‐etiqueta y los programas de pago por servicios ambientales (PES, en inglés), el banco de conservación y el intercambio de hábitats. Resumimos la información sobre las herramientas y exploramos tendencias entre y dentro de los mecanismos basados en criterios que detallan las características geográficas, ecológicas y técnicas de las herramientas. Identificamos 69 herramientas que evaluaron al menos a 34 especies y 39 tipos de hábitat. En donde las herramientas reportaron tarificación, el 98% estaban disponibles gratuitamente. Más herramientas estaban aplicadas a estados ubicados a lo largo de la costa oeste de los Estados Unidos que en cualquier otro sitio y el nivel de transferibilidad de las herramientas varió notablemente entre los mecanismos. Las herramientas comúnmente incorporaron las condiciones a numerosas escalas espaciales, trataron con frecuencia los múltiples riesgos para la viabilidad de sitio y requirieron 1 – 83 entradas de datos. La mayoría de las herramientas requirió un nivel moderado o mayor de habilidad para el usuario. Los estimados medios de la complejidad de las herramientas fueron similares entre todos los mecanismos, a excepción de los programas PES. Nuestros resultados ilustran la diversidad de características ecológicas, necesidades de datos y aplicación geográfica que existe entre las herramientas, lo que respalda las preocupaciones sobre la falta de estandarización. Sin embargo, la consistencia entre las herramientas en cuanto a los requerimientos de habilidades para el usuario, la incorporación de múltiples escalas espaciales y la complejidad resaltan las similitudes importantes que podrían servir como punto inicial para el establecimiento de un desarrollo más estandarizado de herramientas y procesos que incorporen las características del sitio. Una mayor estandarización del diseño de herramientas podría expandir la participación del mercado y facilitar una urgente evaluación de la efectividad de la conservación basada en el mercado.

## Introduction

The United States, like much of the world, is experiencing high rates of biodiversity loss due to habitat fragmentation and destruction (Millennium Ecosystem Assessment 2005; PCAST 2011). Confronting this loss is increasingly prioritized as recognition of biodiversity's contribution to ecosystem and human health and societal and economic well‐being grows (e.g., McShane et al. 2011; Sandifer et al. 2015). Although regulatory mechanisms designed to address biodiversity loss in the United States have been in place for some time (e.g., since 1973 for the Endangered Species Act), market‐based and market‐like (hereafter collectively referred to as market‐based) conservation mechanisms have recently emerged to facilitate biodiversity conservation. Market‐based approaches leverage market forces to more efficiently achieve conservation goals by creating incentives (e.g., revenue for conserving biodiversity) and incorporating environmental externalities in land management decisions (Pindilli & Casey 2015). However, realization of the potential of these markets is hindered by numerous factors, including gaps in available methods for quantifying impacts and benefits to species and habitats (hereafter quantification tools) and lack of transparency and standardization among existing methods (Pindilli & Casey 2015). To address these hindrances, we identified and examined patterns among quantification tools that assess habitat quality or functionality in market‐based conservation in the contiguous United States.

Market‐based conservation mechanisms can be voluntary or compulsory (i.e., regulatory) and may be government sponsored or supported by the private market. For example, the U.S. Department of Agriculture uses cost‐sharing and conservation easement programs (i.e., payments for ecosystem services [PES] programs) that incentivize landowners to voluntarily conserve, restore, or manage important habitats on private lands. Ecolabels, another voluntary practice, rely on consumer preferences and willingness to pay higher prices for goods produced in an environmentally responsible way (e.g., by minimizing negative impacts to species and habitats) (Pindilli & Casey 2015). In contrast, conservation banking is a mechanism underpinned by government regulation requiring compensatory mitigation for impacts to (i.e., incidental take of; United States Congress 1973; USFWS 2012) species listed as at risk, threatened, or endangered. Habitat exchanges, like conservation banks, rely on the creation and purchase of credits to offset impacts to species of conservation concern, though these exchanges operate through voluntary and compensatory means (see Pindilli & Casey [2015] for detailed descriptions and examples of market‐based conservation approaches). Despite differences among these mechanisms, all seek to conserve biodiversity and habitat. To determine the benefits and impacts of development, conservation, and restoration actions, quantification tools are used to substantiate that buyers and investors are getting what they paid for (e.g., viable species and habitats). These tools are central to determining the effectiveness of and guiding conservation and offsetting actions aimed at reducing biodiversity loss (Goncalves et al. 2015).

The effectiveness of tools used in market‐based conservation depends on their scientific soundness (i.e., accurate measurement of benefits and impacts to biodiversity), transparency (e.g., Gardner et al. 2013), and usability (Chen et al. 2013; van Teeffelen et al. 2014). To determine how well a given site contributes to species or ecosystem conservation, the method used to measure a site's functional role needs to be known, understood, and scientifically defensible (Ives & Bekessy 2015). If opaque, a method's accuracy and reliability cannot be evaluated, possibly eroding confidence in its use (Gardner et al. 2013). In conservation banking, market actors are more likely to participate when credit and debit calculations are clear, allowing them to consider costs, revenues, and profitability of a particular action. Such transparency, coupled with accurate and reliable measurements, is also needed to ensure regulators and others have confidence that assessments of biodiversity benefits consistently reflect the contribution of an area to conservation objectives. Finally, the fees, time, and skills required to use an overly complex tool can create transaction costs that negatively impact market performance (van Teeffelen et al. 2014; Pindilli & Casey 2015).

We evaluated quantification tools based on criteria detailing their general, geographic, ecological, and technical features. Our analysis relied on a publicly available database in which 33 criteria describe quantification tools designed for market‐based conservation in the United States (Chiavacci & Pindilli 2018). No previous efforts have sought to gather and assess tools among multiple types of markets. Better knowledge of what tools exist can help practitioners avoid independently developing tools designed to measure the same or very similar systems. Further, because regulators must evaluate the rigor of quantification tools used to meet regulatory requirements, expanding the use of tools already applied, tested, and deemed reliable under various field conditions may streamline regulatory approval. Regarding conservation banking, the lack of both standardization and transparency in quantification tools is a factor needing the most improvement for banking to be more widely adopted as a conservation instrument (Chen et al. 2013; DOI Office of Policy Analysis 2013; Pindilli & Casey 2015).

Our objectives were to support efficient and effective biodiversity and habitat market growth by providing accessible and transparent information about quantification tools used in these markets and insights into ongoing challenges and potential areas for improvement related to these tools. We also considered strategies to improve transparency and standardization among tools.

## Methods

### Tool Identification

We located tools in several ways. We used the Regulatory In‐lieu Fee and Banking Information Tracking System (RIBITS) to identify and collect information on tools used to estimate credits in conservation banking. If RIBITS lacked details on credit estimation methods, we contacted bank managers or points of contact listed on RIBITS to request information about crediting methods. We also sought information from U.S. Fish and Wildlife Service biologists involved in conservation banking to identify other crediting methods. We found additional tools in a review of the literature (e.g., Cochran et al. 2011; Pindilli & Casey 2015) and internet search of tool developer websites (e.g., Environmental Defense Fund and Willamette Partnership). Another important resource was the Ecolabel Index website (http://www.ecolabelindex.com), through which we gathered information about ecolabel program certification criteria. We identified PES programs offered by the U.S. Department of Agriculture through the literature and research efforts mentioned above. To identify tools we may have missed in the literature or internet searches (e.g., tools under development), we used a snowball sampling approach (Goodman 1961), whereby we requested information from market experts and participants regarding obscure tools as well as additional persons to contact.

### Screening

We selected tools to examine based on several conditions. First, tools had to have been designed for use in or have clear applications to biodiversity and habitat markets. For example, we excluded tools with applications to wetland or stream markets unless they had or appeared able to incorporate species components in their quantification of quality or functionality. We also excluded governmental guidance documents that did not specifically incorporate recommendations or methods for quantifying habitat quality or functionality related to species conservation. Second, tools had to be completed or under active development; we excluded incomplete tools lacking a future completion date. Last, we excluded tools replaced by more advanced iterations and examined only the most recent version, even if earlier versions were known by slightly different names.

### Data Collection

We extracted information from documents related to tool application, such as user guides and technical manuals, credit and debit calculators, documents associated with individual conservation banks, species‐ or habitat‐specific conservation plans, peer‐reviewed literature, and guidance documents produced by government agencies. If we could not acquire documents, we asked tool developers to provide details about tool features. We excluded tools if no documents existed and we could not obtain information from tool developers.

### Criteria Development

We developed 33 criteria that encompassed general, geographic, ecological, or technical features of tools (Chiavacci & Pindilli 2018) and used these to guide our extraction of information from tool documents and conversations with tool developers. Criteria development incorporated measurement system assessment standards outlined in Cochran et al. (2011), recommendations from experts and practitioners involved in market‐based conservation efforts, and features we considered important for describing tool functionality. We focused on 16 criteria for the current analysis: pricing, year developed, conservation mechanism, intended users, user skill level, location of use, transferability, number of spatial scales assessed, focal taxa, focal habitat, species presence and abundance, connectivity, risks to site viability, number of data inputs, data input platform, and spatial‐mapping needs (Supporting Information).

General features comprised 5 criteria that conveyed details about a tool's usability, development, and purpose (Supporting Information). The types of conservation mechanisms for which tools were designed included PES programs, ecolabel programs, compensatory mitigation, habitat exchanges, and variable application. We considered habitat exchanges separately from other mechanisms because tools for this mechanism have been developed under compensatory and voluntary contexts and because habitat exchanges represent a unique and emerging mitigation strategy. Unlike habitat exchanges, PES and ecolabel tools were designed strictly for voluntary purposes and the tools we considered as belonging to compensatory mitigation mechanisms were designed strictly for compensatory purposes. We considered a tool to have a variable application when it was not designed for a specific mechanism. We separated intended tool users into agriculture or goods producers and processors, bank sponsors or managers, conservation practitioners, government agencies, Indian tribes, land developers or permittees, landowners or land managers, mitigation program administrators, and technical service providers. We allowed tools to have more than one intended user group. We assigned tools a user skill level of high, moderate, low, or layperson by considering the extent of knowledge and subject matter expertise, technical abilities, and training requirements needed to employ them. For example, a tool requiring an advanced degree, specialized technical skills (e.g., experienced statistical analyst), and expert knowledge of a species or habitat would have a high user skill level. In contrast, a tool requiring no formal training, little or no technical abilities, and no subject matter expertise would have a layperson skill level. If a tool involved different skill levels, we denoted this by listing the 2 levels the skills spanned (e.g., layperson to low skill level). Finally, when determining the year a tool was developed, we used the year listed on tool documents because development sometimes spanned several years or development start and end dates were not listed.

Geographic features comprised 3 criteria describing a tool's broader geographic applicability and spatial extent of assessment (Supporting Information). Regarding the location of tool use, tools developed for use within a specific area of Oregon, for example, were labeled as applying to only Oregon. If a tool could be applied to any state in the contiguous United States, we considered all states as the location of use. We assigned tools a transferability level from low to high based on the number of species, habitat types, and states to which it could be applied. For example, a tool applicable to multiple species, habitat types, and states conveyed a high level of transferability. In contrast, applicability to a single species (or subspecies), habitat type, and state conveyed a low level of transferability. If a tool's features fell into different transferability levels, we denoted this by listing the 2 levels the tool spanned (e.g., low to medium). We quantified the number of spatial scales assessed by tools given that species and habitats are often affected by conditions at multiple scales and because offsetting impacts needs to account for conditions at scales beyond the focal site (Gardner et al. 2013; Goncalves et al. 2015; McKenney & Wilkinson 2015). The spatial scales we counted were only those that factored directly into the estimation of output units (e.g., functional acres and discounted service‐acre years), not those that affected whether a site was approved for protection, restoration, or enhancement. The smallest spatial scale we categorized was the site level and the largest was the species range level.

Ecological tool features comprised 5 criteria describing the scientific information tools incorporated (Supporting Information). We recorded a *yes* or *no* if a tool did or did not, respectively, incorporate some assessment of species presence, abundance, or a similar measure (e.g., density) and if connectivity was or was not assessed from the focal site to surrounding habitats or populations. Connectivity is particularly important to successful conservation (van Teeffelen et al. 2014). We grouped factors addressing risks to site viability into 7 categories because this number of categories encompassed all risks. Categories included the influence of adjacent lands; broader landscape settings; climate change; contamination; habitat loss, degradation, or fragmentation; non‐native or invasive species; or species presence and abundance. Our assessment allowed tools to have more than one type of risk to site viability associated with them. The influence of adjacent lands addressed the condition and uses of lands surrounding focal sites that could negatively impact conditions or populations on the site. Broader landscape settings addressed the relative location of a site within the larger landscape. For example, a tool might assess a site's location within a priority watershed or zone, core of a species range, or other geographically important designation. Climate‐change impacts included either current or potential future effects from a changing climate on a species or habitat. Contamination factors included, for example, the application or presence of contaminants, such as insecticides, herbicides, rodenticides, or fungicides, nutrient leaching or runoff, hazardous materials, or proximity to point‐source polluters. The category addressing habitat loss, fragmentation, or degradation included conditions, such as conversion to nonusable habitat, complete habitat destruction, physical barriers to movement (e.g., roads), or degree of physical disturbance. The non‐native or invasive species category included both plants and animals deemed invasive, non‐native, or both that threaten habitat or population function. The species presence‐or‐abundance category incorporated the condition of a species’ population as an explicit component of viability by determining presence, abundance, density, or a similar measure.

Technical tool features comprised 3 criteria that addressed common data needs and software requirements (Supporting Information). Number of data inputs captured the relative data needs of each tool. The data input platform conveyed programs or file types used for data entry and analysis (e.g., credit calculators in Microsoft Excel). Spatial mapping needs addressed the types of software (e.g., GIS) or mapping requirements tools used to assess spatial features at or around sites.

We generated a tool complexity score to quantitatively summarize a tool's ecological features and needed user skills into a single value. We did this to more easily compare among conservation mechanisms the technical skills and potential amounts of time and data needed to apply a tool. This score included the required user skill level, number of spatial scales assessed, number of risks to site viability assessed, and if connectivity and species presence or abundance were incorporated. For each tool, we assigned points based on each of these features and summed these points to establish complexity scores. Specifically, we scored user skill level from 1 to 7 representing layperson to high skill levels, respectively. We scored spatial scales from 1 to 5 (fewest to largest number of scales assessed, respectively). We scored risks to site viability from 0 to 6 (none to largest number of risks assessed, respectively). Finally, we scored connectivity as 1 or 0 (connectivity was or was not incorporated, respectively) and species presence or abundance as 1 or 0 (presence or abundance was or was not incorporated, respectively). If one of these features was listed as *varies* for a tool (i.e., there was flexibility built into the tool to allow for user‐chosen adaptability), we assigned the lowest possible value for the feature to remain conservative in our estimation. We did not include the number of data inputs for complexity estimates because data inputs for numerous tools varied depending on data availability and regional differences in data requirements and because we had only the maximum number of inputs for some tools. We excluded 7 tools missing information needed to estimate complexity.

## Results

### General Features

**Figure 1:**
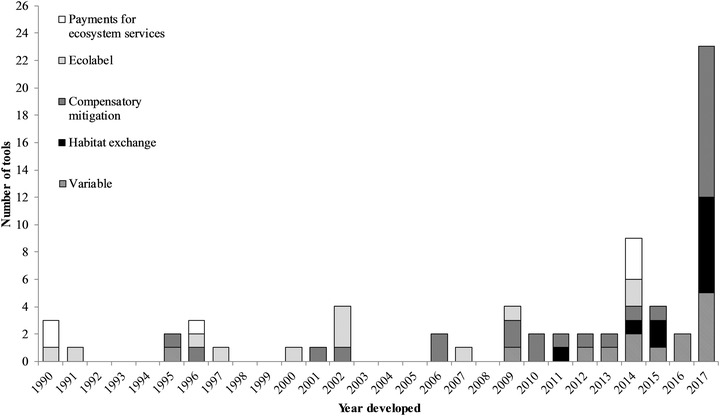
Number of tools developed over time designed to assess habitat quality and functionality for market‐based conservation mechanisms in the contiguous United States, 1990–2017. The number of tools developed within each year is further broken down by the conservation mechanisms for which tools were developed (variable, tools not designed for use under only 1 conservation mechanism). Tools developed in 2017 include 20 that were under development at the time of this analysis.

**Figure 2:**
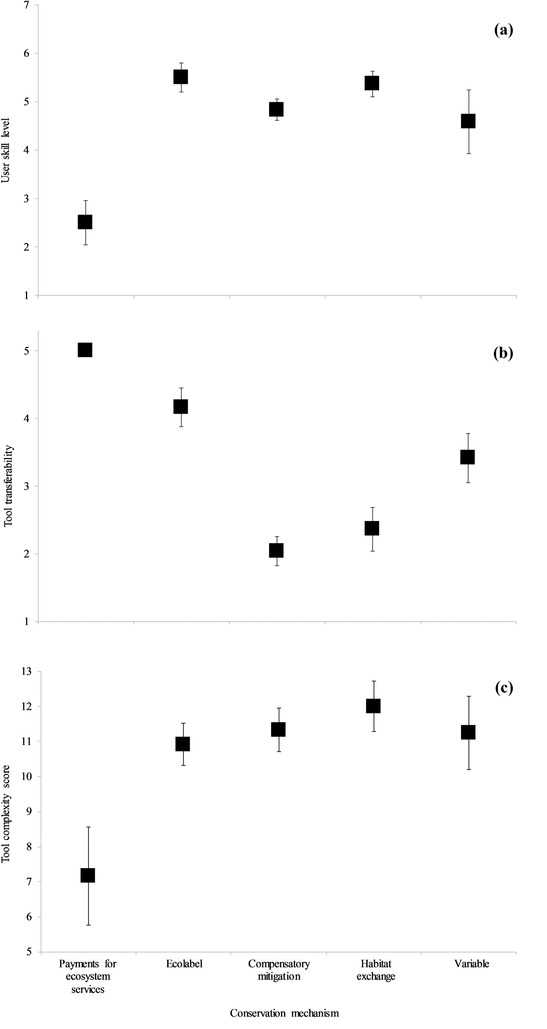
Mean estimates of (a) user skill level, (b) transferability, and (c) complexity scores for tools designed to assess habitat quality and functionality for market‐based conservation mechanisms in the contiguous United States (bars, SE; skill level: 1, layperson; 2, layperson to low; 3, low; 4, low to moderate; 5, moderate; 6, moderate to high; 7, high; transferability: 1, low; 2, low to moderate; 3, moderate; 4, moderate to high; 5, high; complexity, the higher the number, the greater the complexity). Tools are separated by conservation mechanism (x‐axis) (variable, tools not designed for use with only 1 conservation mechanism). All payments‐for‐ecosystem‐services tools had a transferability score of 5 (SE 0).

We identified and examined 69 tools. Twenty‐six tools were designed for use in compensatory mitigation mechanisms, 12 for ecolabel certification programs, 11 for habitat exchanges, and 6 for PES programs. Fourteen tools were not designed for use under a specific mechanism (i.e., tools with variable applications). A mean (SE) of 2.46 (0.53) tools was developed per year from 1990 through 2017 (Fig. 1); 55% were completed (*n* = 18) or started (*n* = 20) since 2014. Of the 63 tools for which pricing could be assessed, 62 were freely accessible in some form and 1 was purchasable. Most tools (*n* = 48, 70%) were designed for multiple user groups, with government agencies (*n* = 38) and landowners and land managers (*n* = 28) being the most common. A majority of tools (*n* = 48, 70%) required a moderate or greater level of user skill, with those for PES programs typically requiring the lowest and those for ecolabel programs and habitat exchanges typically requiring the highest skill levels (Fig. 2a).

### Geographic Features

**Figure 3:**
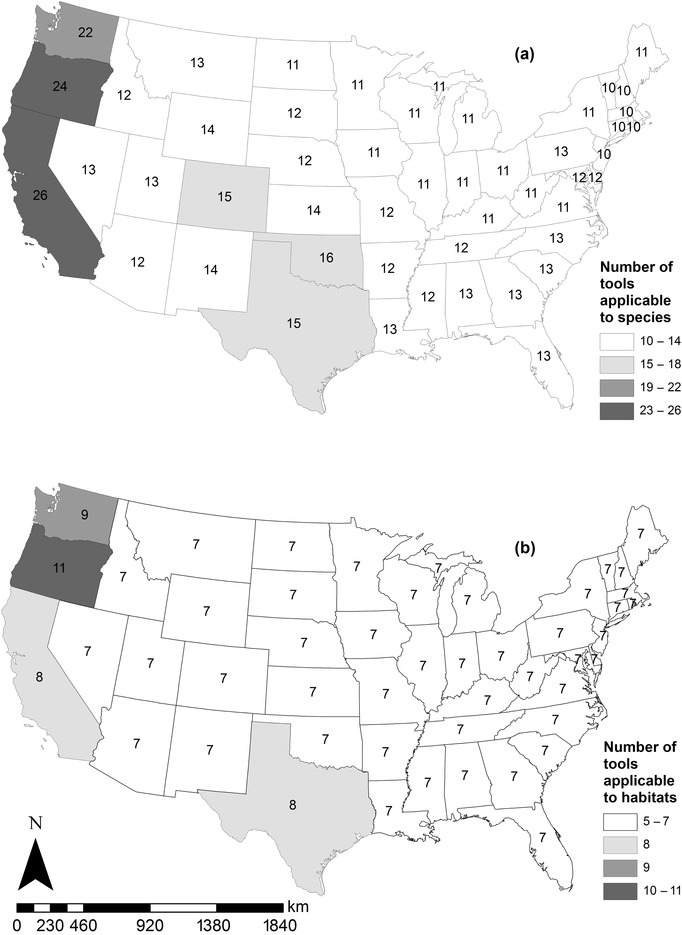
Maps illustrating the number of tools designed to assess habitat quality and functionality for market‐based conservation mechanisms in the contiguous United States applicable to (a) species and (b) habitats in each state.

More tools were developed for use in Oregon, California, Washington, and, to a lesser degree, the south‐central United States than elsewhere (Figs. 3a, b). All PES, 7 ecolabel, 2 compensatory mitigation, and 2 tools with variable applications were applicable throughout the contiguous United States. Excluding these universal tools, those used in habitat exchanges applied to the largest number of states on average (mean [SE] = 4.27 [2.32] states), followed by tools with variable applications (3.83 [1.32]), ecolabel tools (3.00 [0.00]), and compensatory mitigation tools (1.29 [0.12]). Of the 66 tools for which transferability could be assessed, those for PES programs were most transferable, whereas those for compensatory mitigation were lease transferable (Fig. 2b).

### Ecological Features

**Figure 4:**
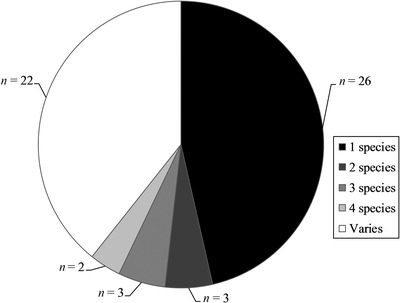
Number of species assessed by individual tools designed to assess habitat quality and functionality for market‐based conservation mechanisms in the contiguous United States (varies, tools designed to assess groups of species or user‐selected species, but that did not list individual species to which tools apply). Thirteen tools that applied to only habitats were excluded.

**Figure 5:**
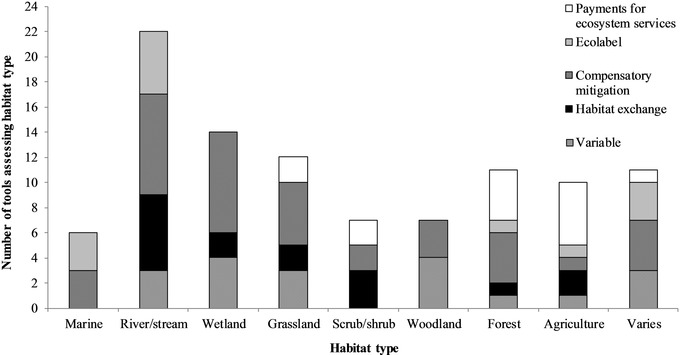
Habitat types assessed by tools designed to assess habitat quality and functionality for market‐based conservation mechanisms in the contiguous United States. Habitat types are further broken down by the conservation mechanisms for which tools were developed (variable, tools not designed for use under only 1 conservation mechanism). Twenty‐eight tools applied to more than 1 habitat type (wetland, wetlands, vernal pools, swamps, floodplains, flooded fields, or other areas that hold freshwater temporarily or permanently but could not be classified as a river, stream, lake, pond, or reservoir; marine, saltwater habitats [e.g., tidally influenced tributaries, intertidal wetlands, subtidal zones, and nearshore ocean habitat]; varies, a tool that could be applied to diverse habitats).

**Figure 6:**
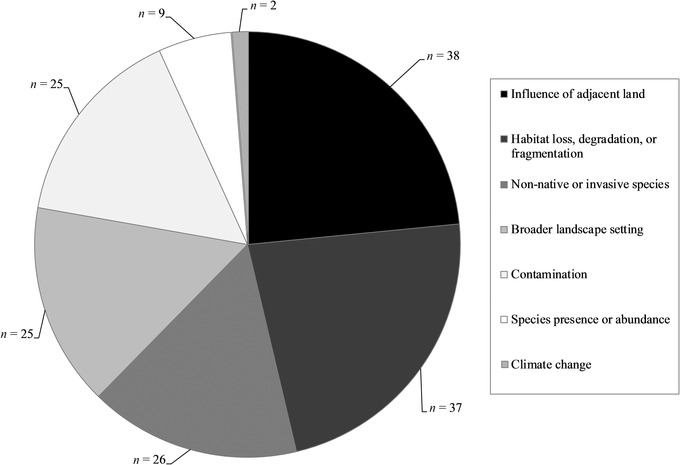
Frequency of different risks to site viability incorporated in tools designed to assess habitat quality and functionality for market‐based conservation mechanisms in the contiguous United States. Risks were categorized into 7 groups and some tools incorporated ≥1 type of risk.

Collectively, tools assessed at least 10 fish, 7 bird, 5 mammal, 3 reptile, 2 amphibian, 4 insect, 1 mollusk, and 2 plant species. Tools most commonly assessed single species or user‐chosen or groups of species (e.g., salmonid species, designated as *varies* in Fig. 4). Ecolabel program tools were commonly designed for groups of species (11 of 12, 92%), whereas most compensatory mitigation (14 of 24, 58%) and habitat exchange tools (9 of 11, 82%) were designed for single species. Many tools (*n* = 39, 57%) assessed multiple habitat types, with rivers and streams being the most common (Fig. 5). Forty‐six (67%) tools incorporated or were capable of incorporating species presence, abundance, or a similar measure and most (*n* = 47, 68%) incorporated habitat connectivity. Among the 56 tools that assessed a fixed number of spatial scales, ecolabel programs incorporated the fewest (mean [SE] = 1.75 [0.17]), followed by variable application (2.58 [0.30]), compensatory mitigation (2.60 [0.23]), and habitat exchange tools (2.73 [0.32]); in all but 1 case, spatial scales incorporated in PES tools varied depending on the circumstances of tool application. Of the 58 tools addressing a fixed number of risks to site viability, 6, 18, 21, 9, 3, and 1 incorporated 1, 2, 3, 4, 5, and 6 risk factors, respectively. The influence of adjacent land was the most common risk factor addressed, whereas the impact of climate change was the least common (Fig. 6).

### Technical Features

The number of data inputs varied highly among tools, with the minimum averaging 20.67 (3.42) (range: 1–83, *n* = 39 tools). Data inputs for 11 tools varied because of, for example, state‐specific (e.g., PES programs) or species‐specific requirements, as well as user customizability. Eleven tools had no data inputs per se because 10 listed only criteria for ecolabel certification and one was a guidance document for establishing and managing mitigation lands. Among the 41 tools that applied data‐input platforms, Microsoft Excel was the most common platform (*n* = 29, 71%), followed by Microsoft Word and PDF formats (*n* = 6, 15%), platforms unique to individual tools (e.g., Visual_HEA, online calculators, *n* = 4, 10%), ArcGIS (*n* = 1, 2%), and a user‐chosen platform (*n* = 1, 2%). Of the 37 tools with identifiable spatial mapping needs, most required a GIS (*n* = 20, 54%) or either a GIS or Google Earth (*n* = 13, 35%), whereas 4 (11%) utilized regionally specific mapping programs (e.g., Oregon Explorer and Landserver).

### Complexity Score

We calculated tool complexity for 61 tools and found variation in complexity within and among conservation mechanisms (Fig. 2c). Tools for PES programs were the least complex, whereas tools for habitat exchanges were the most complex.

## Discussion

Our review of quantification tools developed for U.S. biodiversity and habitat markets highlights how diverse tools are in their spatial and taxonomic application, the ecological factors they incorporate, data needs, and what they require of users. This diversity exists even among tools designed for the same conservation mechanism, due in part to the range of taxonomic‐, habitat‐, and geographically specific applications. Our findings support previous conclusions about the lack of standardization in tool design and function and the array of tools being employed in these markets. Such diversity may increase administrative burden, enlarge costs associated with tool use and approval, and hinder market growth (Bull et al. 2013; van Teeffelen et al. 2014; Pindilli & Casey 2015; Bennett et al. 2017), all of which could hamper the conservation potential and effectiveness of these strategies. By inventorying and describing tool features as we have done, market participants can better determine what tools have been developed, under what situations they apply, where gaps exist in tool availability, and how greater standardization in tool development might be achieved.

Our study revealed increasing tool development since market‐based conservation began in the early 1990s. Although tool development was intermittent through 2009, it has become more pronounced in recent years. This increase appears driven by greater numbers of tools developed for habitat exchanges, conservation banks, and similar mitigation mechanisms and may indicate greater use and appeal of biodiversity and habitat markets (e.g., Mead 2008; Bennett et al. 2017). Increased tool development may also, however, stem from mitigation strategies moving away from assessing credits based on only habitat area (TEEB 2010). Regardless, new tool development is likely to continue if markets continue expanding, heightening the need for more transparency and standardization in tool design.

Usability is a characteristic of a good biodiversity measurement system (Cochran et al. 2011). That many tools did not require highly specialized skills suggests usability should not, in many cases, represent a barrier to tool application. For example, the moderate user skill required by many tools indicates tool developers aimed to balance usability with the scientific and technical expertise expected of users and showed cross‐market alignment in user skill requirements. This pattern in usability is noteworthy considering the intricacies and challenges of measuring habitat and biodiversity (e.g., Bull et al. 2013; Evans et al. 2015). Moderate user skill requirements may also reflect the fact tools were commonly designed for multiple user groups. Another feature complimenting skill requirements was that most tools incorporated familiar and readily accessible software programs (e.g., Microsoft Excel and Google Earth). Our assessment of tool complexity, of which user skill was a component, showed variability within mechanisms, but some consistency across non‐PES mechanisms. The lower complexity of PES program tools was likely necessitated by their need to be applicable to general habitat conditions across large spatial scales, to be used by nonspecialists, and the large number of habitat assessments requiring annual review and ranking. In contrast, tools for non‐PES mechanisms were often designed for certain species or habitats, which may require or allow for more thorough assessments of focal sites and surrounding landscapes. Variation in tool complexity within mechanisms likely arose because of ecological differences among the habitats and species being assessed (e.g., dissimilar impacts of conditions at multiple spatial scales [Wiens 1989]), as well as differences in features prioritized for inclusion due to regulatory requirements and variation in user needs, stakeholders, and regional conservation approaches. For example, tools designed to assess habitats in conservation banking are often unique to each bank (Bennett et al. 2017), producing considerable variation in how credits are quantified. Importantly, tool complexity should not be considered a measure of use difficulty. Assessing use difficulty is subjective because it depends on the user and the user friendliness built into the tool by its designers. Rather, tool complexity represents an estimated measure of the amount of ecological information and user skills needed to use a tool. Similarly, complexity should not be considered a gauge of tool precision. A tool's precision depends largely on the system being measured (e.g., a tool of low complexity may be very precise if high‐quality habitat can be accurately assessed using a few easily measured factors). Studies exploring the relationship between tool complexity and precision would help inform future tool design.

Spatial concentrations of tools across the contiguous United States were very clear, likely stemming from the regional prominence and expansion of conservation mechanisms. Specifically, west coast states, where the largest number of quantification tools applied, have a combination of unique and vulnerable ecosystems and market‐based incentives to protect them (e.g., regulatory, consumer preferences for green products) coupled with land development pressures. Although other regions of the United States, such as the south‐central states, have markets to promote tool development, they are not as numerous as those on the west coast. One potential barrier to market expansion into regions where markets are absent or rare may be the need to develop new tools, a potentially resource‐intensive process. Specifically, existing tools for compensatory mitigation and habitat exchanges are of low transferability because they focus on specific species, habitats, and locations, which limits their use outside small geographic regions. Thus, expanding these mechanisms is likely to require developing new tools or finding a variable application tool of sufficient transferability to meet user, regulatory, and market needs.

The importance of considering conditions on the landscape beyond the focal site during assessments of habitat quality and functionality is broadly recognized with respect to mitigation and other conservation efforts (e.g., USFWS 2003, 2017; Bruggeman et al. 2005; Goncalves et al. 2015). When engaging in mitigation, accounting for the landscape context of a site is important for ensuring ecologically valuable habitats are identified and conserved (Kiesecker et al. 2010; Gardner et al. 2013; Goncalves et al. 2015), although considering only structural links between a site and surrounding landscape may be inadequate for conserving some species (Bruggeman & Jones 2008). For species‐specific tools, deciding how many spatial scales to assess may be dictated partly by species ecology. Furthermore, the fact tools commonly included the influence of adjacent land, broader landscape settings, and connectivity illustrates efforts to include the influence of habitat conditions beyond the focal site. From a site‐viability perspective, integrating conditions across multiple spatial scales shows a concerted effort among and within mechanisms to account for factors potentially affecting the outcome of conservation efforts and the monetary viability of a credit or payment.

Quantification tools are critical to the operation of market‐based conservation strategies. It is, therefore, imperative that market participants can determine what tools exist, what they assess, under what prerequisites they should be applied, and what is required to apply them. Greater transparency and standardization in tool design are essential attributes also noted by others that can help meet these needs (e.g., Bull et al. 2014; Goncalves et al. 2015; Pindilli & Casey 2015; Gamarra & Toombs 2017). Our study, and the tools database on which it was based (Chiavacci & Pindilli 2018), sought to improve the transparency and offer insights into what tool‐related changes might help market performance.

Although it may be infeasible to design a single quantification tool for the diverse species and habitats that could be addressed via market‐based conservation (Bull et al. 2013; Goncalves et al. 2015), standardizing aspects of tool design could provide numerous benefits. Greater standardization could improve consistency in how habitats are assessed, such as before and after sites are altered due to development or restoration activities. It could also make evaluating tools easier for regulators and others, reducing the time and resources needed to approve tools. Such benefits may lower transaction costs or market participants. Familiarity with a standardized design and faster tool approval would offer greater certainty to market participants, such as conservation bankers, seeking to minimize investment costs and risks. Having a standard tool structure could also enable the adaptation of tools to new situations, encouraging transferability, market expansion, and greater inclusion of habitat quality among metrics for some markets (e.g., conservation banking [Gamarra & Toombs 2017]). Several options exist for introducing such standardization. For example, by having tools and their supporting documents (e.g., user manuals, scientific rationale documents, and credit calculators) follow a standardized format for communicating what the tool assesses, how it assesses it, and the rationale behind its design would make this information easier to find, understand, and evaluate. Standard information could include a description of a tool's purpose and scope of application; list of intended tool users and required skills; explanation of where data and professional judgment were used; description of how uncertainty was addressed; information about access to records of decision points made during tool development; details about tool testing; and indication of how and when tool updates will occur. Also, reporting why certain ecological attributes were or were not included in tools would illuminate the decision‐making process. Finally, the adoption of a formalized, expeditious peer‐review process for evaluating tools that can accommodate future tool updates would add a consistent layer of scientific rigor and defensibility to tools.

Biodiversity and habitat markets are lauded for being efficient means of conserving species and habitats (Pindilli & Casey 2015; Bennett et al. 2017) and the increasing number of quantification tools highlights the expansion of these strategies. That similarities already exist among some tools in, for example, user skill requirements, complexity, and spatial scales assessed, suggests that establishing greater standardization in designing and describing tools is not an insurmountable goal. The greater access to information about quantification tools via the recently developed tools database (Chiavacci & Pindilli 2018) and the approaches outlined above for improving transparency and standardization in tool design can inform discussions about advancing the use of biodiversity and habitat markets, given the role of these tools in market operation. Greater transparency and standardization among tools could also help address the widely recognized need for an assessment of the effectiveness of these market‐based strategies at delivering conservation (e.g., Fox & Nino‐Murcia 2005; Carroll et al. 2008; Gamarra & Toombs 2017).

## Supporting information

A list of criteria, including criteria definitions, used to describe and assess patterns in quantification tools (Appendix S1) is available online. The authors are solely responsible for the content and functionality of these materials. Queries (other than absence of the material) should be directed to the corresponding author.
