# Supplementary material for: Trends in biodiversity and habitat quantification tools used for market‐based conservation in the United States
Source: Conserv Biol. 2019 Jul 10;34(1):125–36. doi: 10.1111/cobi.13349 (PMC7027914; doi:10.1111/cobi.13349)
Supplement: Supplementary file 1 — A list of criteria, including criteria definitions, used to describe and assess patterns in quantification tools (Appendix S1) is available online. The authors are solely responsible for the content and functionality of these materials. Queries (other than absence of the material) should be directed to the corresponding author. [file COBI-34-125-s001.doc]

Appendix S1. Criteria used to describe and assess patterns in quantification tools used to measure habitat quality and functionality for biodiversity and habitat markets in the contiguous United States, 1990–2017.

|  | Criterion* | Definition |
| --- | --- | --- |
| General Features | |  |
|  | Pricing | Must user pay to access tool, its documents, or required data or applications |
|  | Year developed | Year tool was first developed |
|  | Conservation mechanism | Conservation mechanism for which tool was designed |
|  | Intended users | Intended users of tool |
|  | User skill level | Level of technical skills and subject knowledge user must possess to apply tool |
| Geographic Features | |  |
|  | Locations of use | States in contiguous United States to which tool applies |
|  | Transferability | Degree to which tool is transferable across taxa, habitats, and geographic locations |
|  | Number of spatial scales assessed | Number of spatial scales incorporated in site assessment |
| Ecological Features | |  |
|  | Focal taxa | Taxa (e.g., species, group of organisms) for which tool was designed |
|  | Focal habitats | Habitats for which tool was designed |
|  | Species presence/abundance | Does tool incorporate species presence, abundance or similar measure |
|  | Connectivity | Does tool incorporate site's connectivity to surrounding habitat or populations |
|  | Risks to site viability | Types of factors that address risks to a focal site's viability |
| Technical Features | |  |
|  | Number of data inputs | Number of individual data inputs required to use tool |
|  | Data input platform | Type of platform into which data are entered |
|  | Spatial mapping needs | Spatial mapping programs/procedures required by tool |

*These criteria (and others not used in this analysis) that describe tool features are in Chiavacci and Pindilli (2018).
